# Morphometric analysis in mouse scleral fibroblasts using focused ion beam/scanning electron microscopy

**DOI:** 10.1038/s41598-019-42758-x

**Published:** 2019-04-19

**Authors:** Kazuhisa Murata, Akira Hirata, Keisuke Ohta, Hiroshi Enaida, Kei-ichiro Nakamura

**Affiliations:** 1 Department of Ophthalmology, Saga University Faculty of Medicine, Saga, Japan; 2 Division of Microscopic and Developmental Anatomy, Department of Anatomy, Kurume University School of Medicine, Kurume, Japan; 3 Hayashi Eye Hospital, Fukuoka, Japan

**Keywords:** 3-D reconstruction, Electron microscopy

## Abstract

The sclera as well as the cornea forms the principal part of the outer fibrous coat of the eye, with a primary function of protecting the intraocular contents and maintaining the shape of the globe. However, the exact morphometric arrangement of scleral fibroblasts remains unclarified. The aim of this study was to observe the three-dimensional structure of the mouse scleral fibroblasts by focused ion beam/scanning electron microscopy (FIB/SEM). Four eyes from C57BL/6J mice were fixed using a mixture of glutaraldehyde and formaldehyde. The sclera was cut out at the equatorial portion and the posterior pole, and postfixed with potassium ferrocyanide, osmium, thiocarbohydrazide, uranyl acetate and lead aspartate. Specimens were then dehydrated and embedded in an epoxy resin. Serial block face images were obtained using FIB/SEM. Three-dimensional image reconstruction and segmentation of the image stack were created using computer software (Amira v6.0.1, FEI). Scleral fibroblasts were arranged in collagenous layers. The cells frequently showed a cellular junction with the neighboring cells and formed cellular networks. Compared with equatorial fibroblasts, there was a more complicated cellular arrangement of the posterior scleral fibroblasts.

## Introduction

The corneal stroma and the sclera form the principal part of the outer fibrous coat of the eye, with a primary function of protecting the intraocular contents and maintaining the shape of the globe. The corneal stroma is a dense connective tissue consisting of regularly arranged collagenous lamellae and fibroblasts known as keratocytes^1–3^. Keratocytes connect to their neighboring cells by gap junctions with fine cell processes and form cellular networks^4–6^. Unlike the cornea, the scleral collagenous lamellae are irregularly arranged. In addition, the scleral fibroblasts were also unevenly organized. Furthermore, previous studies have additionally shown that the scleral fibroblasts are connected to their neighboring cells by gap junctions^5^. However, the exact morphometric arrangement of scleral fibroblasts remains unclarified.

Transmission electron microscopy (TEM) has been used in morphological eye research studies for several decades. However, the obtained images from thin sections often fail to interpret the three-dimensional arrangement of the cellular components. The use of serial sections for TEM is one approach that has been used to overcome this problem. Even so, this method remains a lengthy and tedious process. Recently, an alternative to the serial section TEM approach for imaging tissue has been described for scanning electron microscopy (SEM). With this approach, a resin-embedded tissue sample is serially sectioned using a focused ion beam (FIB) directed parallel to the block face for milling thin layers of the embedded tissue^6,7^. The milled face is then imaged using the scanning electron beam. Since this technique can be highly automated, this removes many of the problems associated with the manual serial section manipulation and imaging in TEM. As a result, the process is able to automatically generate aligned serial images.

Here, we used SEM equipped with a FIB (FIB/SEM) to investigate the three-dimensional structure of the normal scleral fibroblasts in mice. The aim of this study was to clarify the cellular arrangement in scleral fibroblasts.

## Results

**Figure 1:**
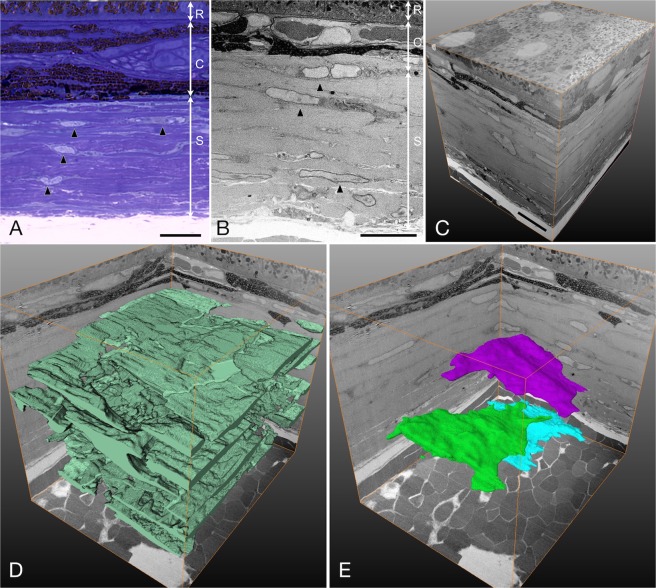
Cross-sectional image and three-dimensional structure of the sclera at low magnification. (**A**,**B**) Light micrograph from a toluidine blue-stained resin section (**A**) and a FIB/SEM image of the sclera at the posterior pole (**B**). Spindle-shaped scleral fibroblasts are arranged between the collagenous layers (arrowheads). Bars, 10 µm. R, retinal pigment epithelium; C, choroid; S, sclera. (**C**) Three-dimensional view of the stack of the 600 serial block surface images of the sclera at the posterior pole, 30 µm × 50 µm × 60 µm. Bar, 10 µm. (**D**) Three-dimensional view of the scleral fibroblasts outlined by segmentation. Green, scleral fibroblasts (**E**) Three selected fibroblasts showing a polygonal shape and flattened cytoplasm.

In the cross sections, the sclera at the posterior pole was thicker than that at the equatorial portion (34.01 ± 1.23 µm vs. 20.48 ± 1.68 µm, p = 0.0014, paired t-test, Table 1, Fig. 1). The scleral fibroblasts were located among multiple collagenous layers and appear to have a spindle shape with thin and fine processes (Fig. 1A,B). A stack of serial images formed a three-dimensional reconstruction image of the sclera (Fig. 1C). Segmentation of whole scleral fibroblasts revealed that the cells spread out within the collagenous layers, and were arranged as a reticular network (Fig. 1D). Each fibroblast was spread among the collagenous layers and showed a polygonal shape and flattened cytoplasm parallel to the scleral wall, except for the areas surrounding the cell nuclei (Fig. 1E). The total volume that the scleral fibroblasts occupied on scleral stroma at the posterior pole was 18.62 ± 1.61% and 16.60 ± 1.60% at the equatorial portion (Table 1). There was no significant difference between the groups (p = 0.34, paired t-test). The mean volume of each scleral fibroblast at the posterior pole was 714.57 ± 76.94 µm^3^, while that for each scleral fibroblast at the equatorial portion was 704.09 ± 93.20 µm^3^. There was no significant difference between the groups (p = 0.91, paired t-test, Table 1).

**Table 1:** Scleral thickness, cell density, cell size and number of cell junctions.

	Posterior pole (n = 4)	Equatorial portion (n = 4)	p-value
Scleral thickness (mean ± SE µm)	34.01 ± 1.23	20.48 ± 1.68	0.0014
Cell density (mean ± SE %)	18.62 ± 1.61	16.60 ± 1.60	0.34
Cell volume (mean ± SE µm^3^)	714.57 ± 76.94	704.09 ± 93.20	0.91
Number of cell junctions per cell (mean ± SE)			
total	5.01 ± 0.34	4.90 ± 0.64	0.89
horizontal	2.64 ± 0.24	3.43 ± 0.82	0.39
vertical	2.37 ± 0.11	1.47 ± 0.24	0.029

The values are presented as the mean ± standard error of the mean (SE).

**Figure 2:**
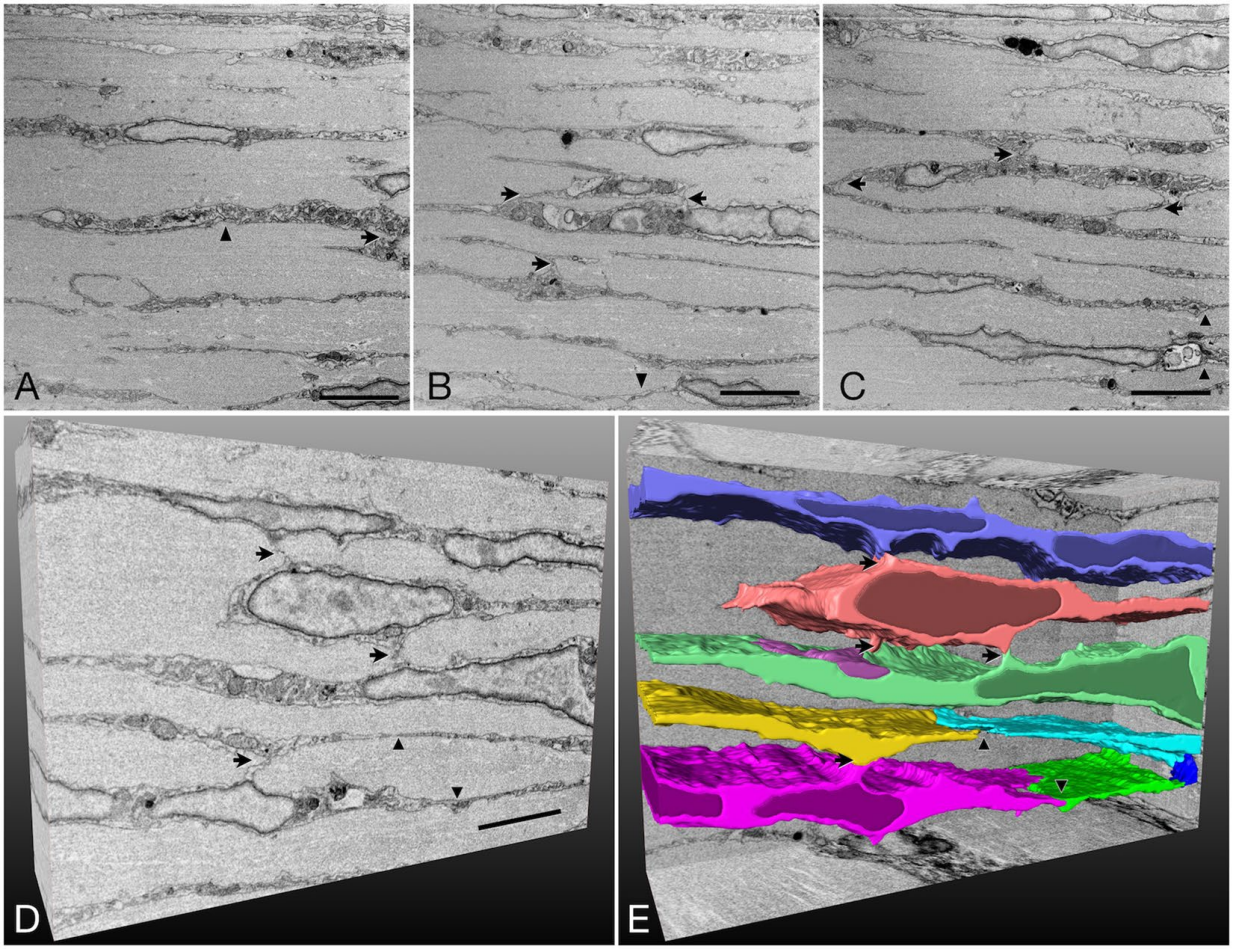
Block surface images and three-dimensional structure of the sclera at the posterior pole showing cell junctions. Note horizontal (arrowheads) and vertical (arrows) junctions. (**A**–**C**) Sections of the scleral stroma at an interval of 10 µm. Spindle-shaped scleral fibroblasts are arranged between the collagenous layers. Bars, 5 µm. (**D**) Three-dimensional view of the stack of the images. Bar, 5 µm. (**E**) Seven selected fibroblasts partially outlined by segmentation for 100 serial block surface images at 50-nm intervals in the z plane images showing horizontal (arrowheads) and vertical (arrows) junctions.

**Figure 3:**
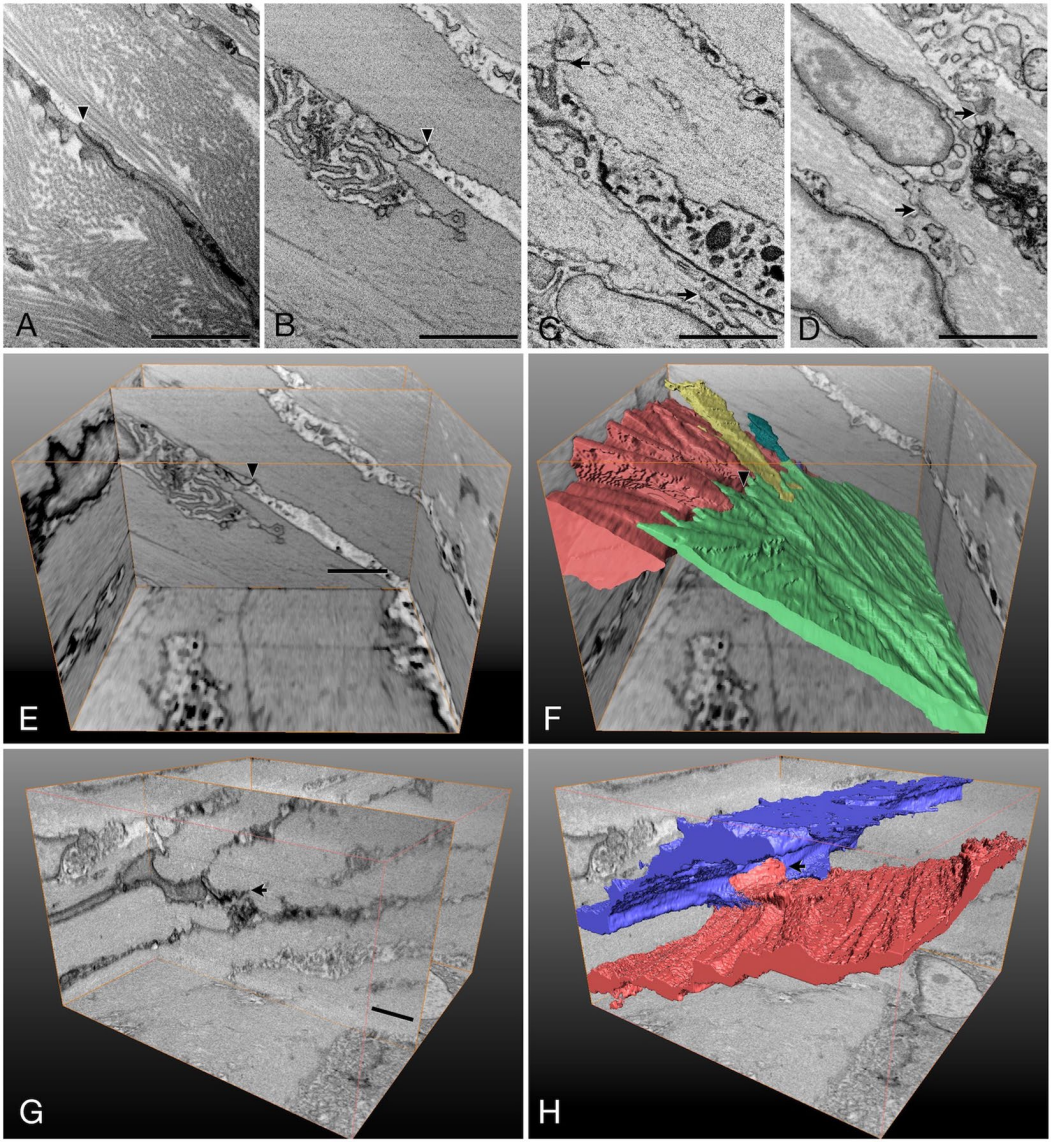
High magnified images of the scleral fibroblasts showing cell junctions. (**A**–**D**) Block surface images of the scleral stroma at the posterior pole (**A**–**C**) and at the equatorial portion (**D**). Note the horizontal (arrowhead) and vertical (arrows) junctions. Bars, 2 µm. (**E**) Three-dimensional view of the stack of images of the 400 serial block surface images at the posterior pole, 8 µm × 6 µm × 20 µm, showing the horizontal junction (arrowhead). Bar, 2 µm. (**F**) Three-dimensional view of scleral fibroblasts outlined by segmentation at the same area of (**E**). Note the wide range of the cell junction (arrowhead). (**G**) Three-dimensional view of the stack of the images of the 300 serial block surface images at the equatorial portion, 8 µm × 6 µm × 15 µm, showing the vertical junction (arrow). Bar, 2 µm. (**H**) Three-dimensional view of the scleral fibroblasts outlined by segmentation at the same area of (**G**). Note the vertical cell junction (arrow).

The cells were connected to the neighboring cells both horizontally and vertically (Figs 2 and 3). In the block surface images, the fine cell processes seemed to form attaching sites horizontally. Vertical attachments were formed by the cell body and cell processes. At the posterior pole, the scleral fibroblasts were connected to the neighboring cells horizontally with 2.64 ± 0.24 sites/cell and vertically with 2.37 ± 0.11 sites/cell. On the other hand, at the equatorial portion, the cells were connected to the neighboring cells horizontally with 3.43 ± 0.82 sites/cell and vertically with 1.47 ± 0.24 sites/cell. Although there was no significant difference in the number of total and horizontal cell junction sites, the number of vertical junctions in the posterior sclera was more extensive than that in the equatorial sclera (p = 0.029, paired t-test, Table 1). The junctional sites were classified into five groups (Table 2). More than 90% of the horizontal junctions were formed by cell processes with the area of 1.44 ± 0.20 µm^2^ (Table 2). In the vertical junctions, cell body-cell process junctions were predominant with the area of 2.29 ± 0.43 µm^2^ (Table 2). Most of the connected sites exhibited the close apposition of the cell membranes without an identifiable specific junction structure (Figs 2 and 3).

**Table 2:** Area of cell junctions.

	Horizontal junctions		Vertical junctions			p-value
	Hbb (n = 4)	Hpp (n = 43)	Vbb (n = 9)	Vbp (n = 29)	Vpp (n = 15)	
Area of junctions (mean ± SE µm^2^)	12.15 ± 4.45	1.44 ± 0.20*	5.93 ± 1.09	2.29 ± 0.43	0.76 ± 0.14^†^	<0.0001

The values are presented as the mean ± standard error of the mean (SE).

Hbb, horizontal cell junctions formed between cell bodies; Hpp, horizontal cell junctions formed between cell processes; Vbb, vertical cell junctions between cell bodies; Vbp, vertical cell junctions formed between cell body and cell process; Vpp, vertical cell junctions formed between cell processes.

*Significant difference from Hbb; ^†^Significant difference from Vbb.

**Figure 4:**
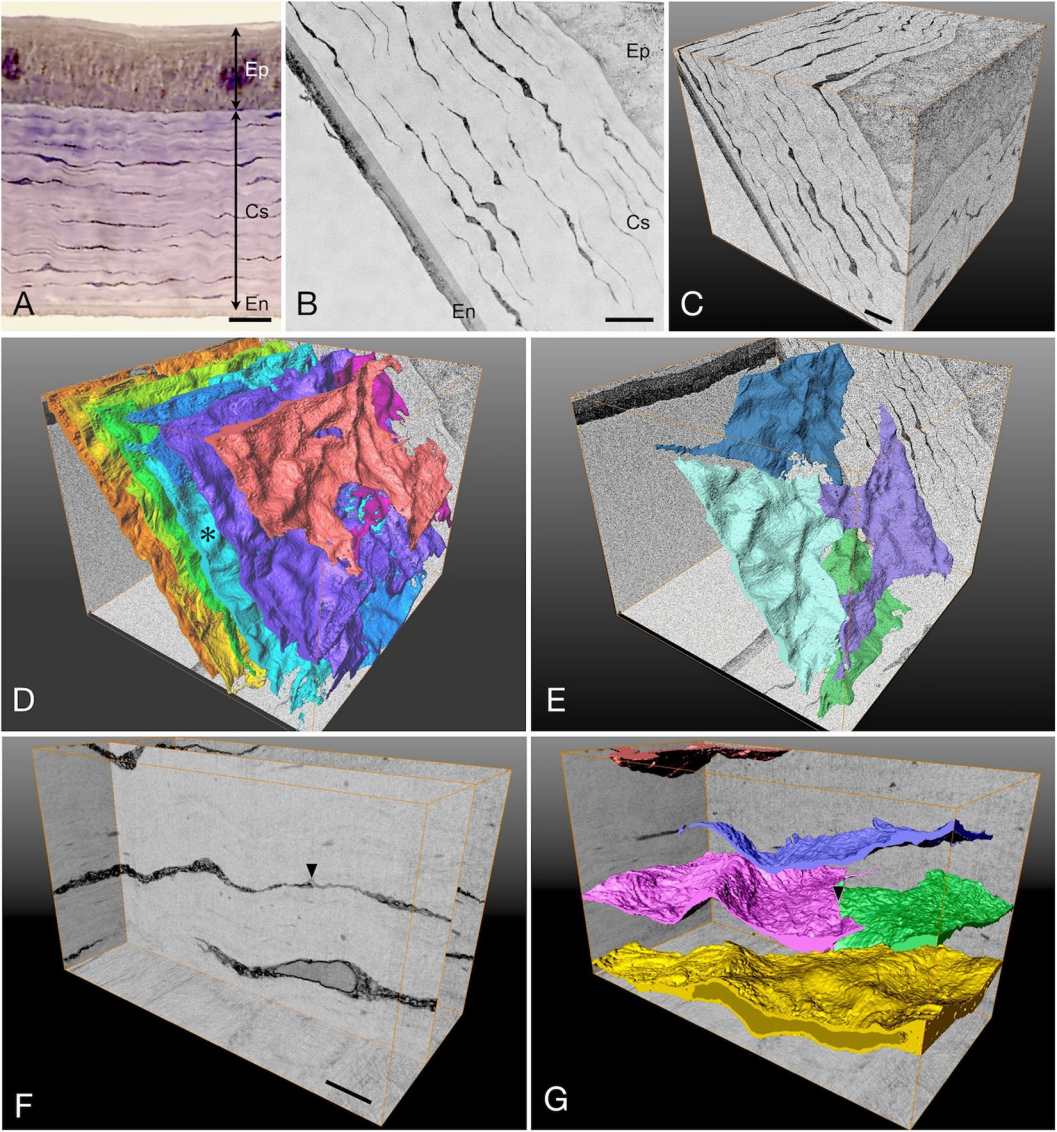
Cross-sectional image and three-dimensional structure of the cornea. (**A**) Light micrograph from a toluidine blue-stained resin section of the of the cornea. Thin and spindle-shaped keratocytes are arranged between the thick collagenous layers. Bar, 10 µm; Ep, corneal epithelium; CS, corneal stroma; En, corneal endothelium. (**B**) FIB/SEM image of the of the cornea. Bar, 10 µm; Ep, corneal epithelium; CS, corneal stroma; En, corneal endothelium. (**C**) Three-dimensional view of the stack of the 880 serial block surface images of the cornea, 70 µm × 80 µm × 88 µm. Bar, 10 µm. (**D**) Three-dimensional view of the keratocytes outlined by segmentation at the same area of (**C**). Each cell layer is categorized by different colors. (**E**) A single cell layer (sky blue-colored layer indicated with the asterisk in (**D**) is segmented and shows that the layer is composed of 4 flattened keratocytes. (**F**) Three-dimensional view of the stack of images of the 200 serial block surface images of the keratocytes, 5 µm × 8 µm × 10 µm. Note the cell junction (arrowhead). Bar, 1 µm. (**G**) Three-dimensional view of the keratocytes outlined by segmentation at the same area of (**F**). Note the broad range of the cell junction (arrowhead).

To fully understand the orientation of the scleral fibroblasts, a comparison of the keratocytes in the corneal stroma with the scleral fibroblasts was performed. The corneal keratocytes were located among well-ordered, thick, multiple collagenous layers and appeared to have a spindle shape with thin and fine processes (Fig. 4A–C). In the three-dimensional view, the cells spread out between the collagenous layers, and connected with the neighboring cells to form a cellular plane (Fig. 4D,E). These cells were connected horizontally with broad areas of other cells, rather than by focal junctions with cell processes (Fig. 4F,G). There were few vertical connections. The cellular density of the keratocytes was 7.24% ± 0.82% (n = 4 eyes), which is significantly smaller than that of the scleral fibroblasts at the posterior pole with 18.62 ± 1.61% (p = 0.0007, paired t-test).

## Discussion

In this study, we used FIB/SEM to examine the three-dimensional ultrastructure of the scleral fibroblasts in mice. Mice are widely used in ophthalmic research as a model of several disorders, such as glaucoma and myopia^8,9^. Although many studies have revealed the structures of the scleral fibroblasts, most studies were performed by examining cross sections with both light and transmission electron microscopy. However, there have been no reports describing their three-dimensional organization using the serial sectioning method. In order to understand the nature of the scleral fibroblasts, it is necessary to observe the intact sclera with the current method using FIB/SEM. FIB/SEM results revealed that the scleral fibroblasts came in contact with the neighboring cells via both the horizontal and vertical direction to form a complicated cellular network. In addition, the areas of the cell contact were relatively broad as opposed to having focal contact with fine cell processes.

In previous reports, the three-dimensional arrangement of corneal keratocytes was well documented^10,11^. Nishida *et al*. reported that by applying the denudation method, which selectively removes the extracellular connective tissue components, they were able to observe a three-dimensional network of the corneal keratocytes^10^. This study reported that the keratocytes were stellate with short and often branched processes. In contrast, the current study demonstrated that the keratocytes had more of a polygonal shape rather than a stellate shape with narrow intercellular space. The differences between the two studies are probably due to variations in specimen processing and the degree of shrinkage during specimen preparation. As the procedure of specimen processing used in this study seems suitable for enhancement of the membrane contrast and reducing the amount of tissue shrinkage^12^, the morphometric data presented here reflect the actual data for living tissues. Besides, Poole *et al*. found that the shape of the keratocytes differed according to the location of the keratocytes, with the cells in the posterior cornea having large cell bodies and extensive short processes that connected with the neighboring keratocytes^13^. In addition, the network of keratocytes in the cornea is thought to be involved in the stromal metabolism, inflammatory reactions and protection against infection via cellular communication^14^.

Compared with corneal keratocytes, our current study demonstrated that the scleral fibroblasts exhibited a complicated arrangement of the fibroblast network. The cells were more of a polygonal shape with cell processes, and they were connected to the neighboring cells with around five sites. At the posterior pole, the fibroblasts were found to more frequently attach to the neighboring cell in the vertical direction. This may reflect a regional difference in the scleral metabolism. Moreover, the cellular density of the scleral fibroblasts was more abundant than the cellular density of the corneal keratocytes. This appears to indicate that the scleral fibroblasts have a more active role in the scleral metabolism than previously expected.

In the present study, the exact types of cell junctions could not be elucidated. The FIB/SEM method is suitable for the correlative seamless observation of specimens from the light microscopy to the TEM level, which is a valuable approach to understanding the cell and tissue organization of organisms^15^. In addition, three-dimensional reconstruction methods using block face imaging are powerful tools for understanding the mesoscale architecture of cells and tissues, since they provide detailed three-dimensional information comparable to the serial sectioning method of TEM, and they also provide a larger volume of information than the electron tomography method^16^. On the other hand, the limitation of this method is the resolution of the images obtained, especially in low magnification. The technological progress in image processing speed and the detecting device could overcome this issue in the future.

Raviola *et al*. reported that scleral fibroblasts had gap junctions, tight junctions and intermediate junctions by thin-section electron microscopy and freeze fracture analysis methods^5^. In a recent study, connexin 43, a gap junction protein, was found in scleral tissue in healthy and pathologic conditions^17,18^. Together with the results of the current study, these findings suggest that the scleral fibroblasts might be additionally involved in the regulation of scleral metabolism, as well as protecting the intraocular contents and maintaining the shape of the globe.

Fibroblast networks have also been found in several tissues, including the cornea, digestive organs, heart and uterus, and thus may be involved in organ function^11,19–21^. Langevin *et al*. speculated that fibroblasts form an extensively interconnected cellular network, which suggests that they may have important and as of yet, unsuspected integrative functions at the level of the whole body^22^.

In conclusion, the findings of the current study revealed the presence of a three-dimensional arrangement of the fibroblasts in the sclera. As far as we know, this is the first report of a three-dimensional arrangement of the scleral fibroblasts observed with FIB/SEM.

## Materials and Methods

### Animals

All experiments were performed in accordance with the National Institutes of Health Guidelines for animal research and with the ARVO Statement for the Use of Animals in Ophthalmic and Vision Research. All animal procedures were approved by the Board for Animal Experiments of Saga University. The study used four right eyes of four six-week-old male C57BL/6J mice (KBT Oriental Co., Ltd., Saga, Japan).

### Specimen preparation

After mice were euthanized by carbon dioxide inhalation, eyes were enucleated and immersion fixed in a mixture of 2.5% glutaraldehyde, 2% paraformaldehyde and 2 mM CaCl_2_ in 0.1 M cacodylate buffer (pH 7.4) for 2 h. The eyes were cut circumferentially at the limbus in order to separate the anterior cornea and posterior sclera. The specimens were further fixed in the same fixative for 2 h at 4 °C. The scleral specimens were cut out at the posterior pole and at the equatorial portion to construct a 2 × 2 mm square piece. As a control, the corneal specimens were also cut out at the center of the cornea with a 2 × 2 mm square piece. In order to enhance the membrane contrast and to reduce the amount of tissue shrinkage, the specimens were then postfixed using a combination of the ferrocyanide-reduced osmium method and the osmium-thiocarbohydrazide-osmium method as previously described^12,23–26^. Briefly, after five washes in the cacodylate buffer, the specimens were postfixed for 1 h in a solution containing 2% osmium tetroxide and 1.5% potassium ferrocyanide in the cacodylate buffer at 4 °C. The specimens were then washed five times with distilled water and immersed in 1% thiocarbohydrazide solution for 1 h. After five washes with distilled water, the specimens were further immersed in 2% osmium tetroxide in distilled water for 1 h and then washed five times with distilled water. Subsequently, the specimens were en bloc stained in a solution of 4% uranyl acetate dissolved in distilled water overnight for contrast enhancement. After the staining, the specimens were washed with distilled water, and then further stained by Walton’s lead aspartate solution for 2 h^27^. Next, the specimens were dehydrated in an ethanol series, followed by infiltration of the epoxy resin mixture (Epon 812, TAAB, Berkshire, England), and polymerized for 72 h at 60 °C. The surfaces of the embedded specimens were cut vertically to expose cross sections using a diamond knife on an Ultracut E Microtome (Leica, Wetzlar, Germany). The resin blocks were then placed on a standard SEM microscope specimen holder with adhesives for SEM imaging.

### FIB/SEM tomography and three-dimensional structure reconstruction

The freshly exposed surface of the specimens was examined by backscatter electron imaging using a conventional field emission SEM with FIB (FIB/SEM, Quanta Three-dimensional FEG, FEI, Eindhoven, The Netherlands). To prevent charging, the specimens were coated with a thin layer of evaporated carbon^15^. Serial images of the block face were acquired by repeated cycles of sample surface milling and imaging using the Slice & View G2 operating software (FEI). Milling was performed with a gallium ion beam at 30 kV with a current of 15 nA. The milling pitch was set to 50 nm/step and 800–1000 cycles for the high magnification images, and to 100 nm/step and 600 cycles for the low magnification images. The images were acquired at the landing energy of 3 keV with a bias voltage of 1.5 kV. The resultant image stack was processed using the ImageJ (https://imagej.nih.gov/ij/) and Amira v6.0.1 software (FEI Visualization Science Group, Burlington, MA). To observe the three-dimensional structures of the scleral fibroblasts, the membrane components of the cells were semi-automatically segmented. Subsequently, the outlined cells were then visualized and displayed.

### Statistical analysis

The scleral thickness was measured at the posterior pole and at the equatorial portion and calculated as the mean from 20 randomly selected images. The cellular densities of the scleral fibroblasts and corneal keratocytes were calculated from the obtained images in each specimen as the total cell volume divided by that of the total scleral stroma or corneal stroma. The values are presented as mean percentages (%) ± standard error of the mean (SE). The morphometry of the scleral fibroblasts including cell volume and the number of the cell junctions was measured and calculated from the obtained images in 6 cells in each specimen. The values are presented as the means ± SE from four specimens. The areas of cell junctions were measured at the posterior pole and at the equatorial portion at 100 consecutive sites (15 each at the posterior pole from the 4 specimens and 10 each at the equatorial portion from the 4 specimens). According to the direction of the junctional sites and its shape, they were classified into five groups; horizontal cell junctions formed between cell bodies (Hbb), horizontal cell junctions formed between cell processes (Hpp), vertical cell junctions between cell bodies (Vbb), vertical cell junctions formed between cell body and cell process (Vbp) and vertical cell junctions formed between cell processes (Vpp). The values are presented as the mean ± standard error of the mean (SE).

Statistical analyses of the differences were performed using computer software (GraphPad Prism 5 for Macintosh; GraphPad Software Inc., La Jolla, CA). The differences in scleral thickness, cell volume, and number of cell junctions in the two locations of the scleral fibroblasts, and the difference in the cellular density of the scleral fibroblasts at the posterior pole and the corneal keratocytes were evaluated using paired *t*-tests. The differences of the area of cell junctions among the five groups were performed using one-way ANOVA. When significant differences were observed, a Tukey’s multiple comparisons test was performed for between-group comparisons. P-values less than 0.05 were considered to be significant.
